# Common injuries and risk factors in South African long-distance runners over a 12-month period

**DOI:** 10.17159/2078-516X/2026/v38i1a22710

**Published:** 2026-09-15

**Authors:** T Nadar, L Lategan

**Affiliations:** Department of Sport and Movement Studies, University of Johannesburg, South Africa

**Keywords:** Overuse injury, prevention, running, causes, lower limb, knee

## Abstract

**Background:**

The 12-month incidence of injuries in long-distance running is high, and injury rates vary between 17% and 90%.

**Objectives:**

The aim of this study was to describe injury incidence, type, location and risk factors in long-distance runners over the last 12 months.

**Methods:**

A retrospective, descriptive, quantitative research design was used. Participants (n=215) were drawn from South African long-distance runners. A previously used online one-year injury recall questionnaire was used to investigate injury incidence, type, location and risk factors. Descriptive and inferential statistics were used to develop a model explaining the risk of injury in runners based on significant risk factors. Statistical significance was set at p<0.05.

**Results:**

In total, 157 injuries were reported by 110 runners (51%). When comparing by sex, 54% of males and 48% of females reported an injury. The sex comparison also showed that males ran significantly faster than females (p=0.04). Most injuries were due to overuse (79%). Common injury locations included the knee (26%), the ankle/foot complex (19%), and the lower leg (18%). There was no significant difference in injury location between male and female runners (p=0.13). Significant injury risk factors identified included: low BMI (p=0.002), greater height (p=0.05), lack of running experience (p=0.05), not warming up (p<0.001), not cooling down (p=0.01), not stretching before running (p=0.004), not stretching after running (p=0.01), and previous injury (p<0.001). Logistic regression analysis, using only the significant injury risk factors, yielded an injury prediction accuracy of 78% (p<0.001).

**Conclusion:**

The 12-month incidence of injury in long-distance runners was 51%, and several significant risk factors were identified. These risk factors explained or predicted 78% of the injuries sustained. Most injuries were due to overuse and occurred in the lower limbs. In the current sample of runners, not stretching and not warming up prior to running were statistically significant risk factors for overuse injuries.

Long-distance runners are exposed to repetitive impact forces, particularly during prolonged sessions, which can lead to overuse injuries. Runners have a high risk of getting injured, with incidence rates ranging from 2.5 to 33 injuries per 1000 hours of running exposure.^[1]^ Hendricks and Phillips^[2]^ reported an injury incidence rate of 0.67 per 1 000 km run in long-distance club runners, while Hespanhol Junior et al.^[3]^ reported a rate of 10 injuries per 1 000 hours of running exposure in recreational runners.

In 2007, van Ghent et al.^[4]^ conducted a literature review and reported that the 12-month injury incidence among long-distance runners ranged from 17% to 90%. Franke and colleagues^[5]^ reported that 52% of runners sustained an injury in the previous 12 months. Furthermore, they observed that 40% of uninjured runners sustained a new running-related injury during a 16-week preparation period for either a half-or a full marathon. This finding was subsequently confirmed by Kakouris and co-workers^[6]^, who also reported an injury incidence rate of 40% in long-distance runners. In addition, Sanfilippo and co-workers^[7]^ surveyed 3 669 runners and found that 51% reported an injury in the previous 12 months. Data on South African runners is scarce; however, Ellapen et al.^[8]^ reported an injury incidence of 90% in recreational half-marathon runners from the KwaZulu-Natal Province (South Africa). Furthermore, Hendricks and Phillips^[2]^ reported that 32% of club runners in Cape Town (Western Cape Province, South Africa) sustained an injury, but they monitored the runners only over a 16-week period.

Although the majority of running-related injuries occur at the lower limb, some authors have also reported lower back injuries in long-distance runners.[6,8] There is general consensus that the most common running-related injuries occur at the knee, lower leg and ankle-foot complex.[6,9] Ellapen, Satyendra, Morris, and Heerden[8] reported similar findings in South African long-distance runners: knee (26%), tibia/fibula (22%), and lower back/hip (16%). Tendinopathies, stress fractures, and muscle strains account for most injuries in long-distance runners.^[2]^

Risk factors for running-related injuries are varied and widely debated. Although there is some consensus that a previous injury is a significant risk factor for a recurrent or new injury in runners,^[3,7,10]^ there is less agreement on the following proposed running-related injury risk factors: older age,^[10]^ being a novice runner,^[11,12]^ inadequate muscle strength or muscle imbalance, higher weight/body mass index (BMI),^[7,10]^ stretching before running, and irregular and/or absent menstruation in female runners.^[7,9]^

To date, there is no consensus on the significant injury risk factors in long-distance running. Therefore, this study’s main objectives were to describe injury incidence, type, location and significant injury risk factors in long-distance runners. This study also analysed differences in these objectives between male and female long-distance runners.

## Methods

### Study design and ethical considerations

A retrospective, quantitative, descriptive research design was used. Participants were recruited through online running forums and social media platforms, resulting in a sample of 215 long-distance runners (50.7% male, 49.3% female). Inclusion criteria were long-distance runners (running at least 3 times per week and completing 10 km or more in a single run at least once a month), aged 18 years or older, registered road runners in South Africa, male or female, and with a minimum of 6 months of running experience. Exclusion criteria included running fewer than 3 times per week, consistently running less than 10 km in a single run, and playing any other sport at a competitive level. Prior to data collection, ethical approval was granted by the University of Johannesburg’s Health Science Research Ethics Committee under clearance number REC-2040-2023.

### Data collection

Data were collected through a previously used online questionnaire that included sections to extract demographic information, training habits, and injury history over the previous 12 months.^[3]^ The injury section of the questionnaire was based on injury type, location, severity and whether the injury was new or recurrent. The following definition was used for participants to identify whether they had sustained an injury or not: “any physical complaint that affected participation in normal training or competition, led to reduced training volume, experience of pain or reduced performance in sports”.^[23]^

### Data analysis

Descriptive and comparative analyses were utilised. Inferential statistics, including independent-samples tests and effect sizes, were used to identify significant injury risk factors, and a logistic regression analysis of these factors was used to predict injury incidence. The level of statistical significance was set at 95% (p<0.05).

## Results

### Participant demographics

The sample comprised 215 long-distance runners (51% male, 49% female). Although male runners were significantly taller (p=0.001) and heavier (p=0.001) than female runners, their BMI values were similar (p=0.26) (Table 1). Most runners (72%) had more than two years’ running experience (Table 2). On average, they trained 4.3 times per week, covered 40.4 km per week, ran at 6.6 min/km and spent 68.6 minutes per training session. Male runners ran significantly faster during training than female runners (p=0.04) (Table 3). Most runners (64%) reported that their motivation for running was “health improvement” and “achieving their best performance”.

### Injury incidence

The injury incidence over 12 months was 51% (Table 4). There was no significant difference (p=0.38) in injury incidence between male (54%) and female runners (48%). Many runners (60%) reported a new injury, while 40% reported a recurring injury. Most injuries were overuse injuries (79%). The most common injuries were musculotendinous or musculoskeletal injuries (79%). The injury locations included the knee (26%), ankle/foot complex (19%), the lower leg (19%), the hip and pelvis (14%), thigh (12%), calf (7%), spine (2%), and the upper limbs (1%).

In terms of severity, most injuries were either moderate (58%) or minor (29%), with only 12% being severe.

### Injury risk factors

Injury risk factors were analysed for significance (Table 5). The significant injury risk factors identified were a lower BMI (p=0.002), greater height/stature (p=0.05), lack of running experience (p=0.05), not warming up (p<0.001), not cooling down (p=0.01), not stretching before running (p=0.004), not stretching after running (p=0.01), and having suffered a previous injury (p<0.001). Through logistic regression analysis (using the abovementioned significant injury risk factors) it was possible to identify injury incidence with an association of 77.8% (p<0.001).

## Discussion

The total injury incidence over the 12-month study period of 51% is similar to past research findings reporting values ranging from 16% ^[13]^ to 85%.^[14]^ Van Poppel, Scholten-Peeters, van Middelkoop and Verhagen^[15]^ report a similar injury incidence of 46.3% during a 12-month period of running. Another South African study has reported a 12-month running injury incidence of 30%.^[2]^ The 51% injury incidence found in this study may seem high; however, injury exposure is high in runners; the average runner ran more than 40 km per week, which accumulated to more than 2 000 km per year. Other sports also have equally high injury incidence, including racquet sports (54%), combat sports (64%), and team sports (75%).[16] Sports that had lower injury incidence compared to the present study, include football (38%), strength training (9%), handball (39%), and tennis (18%).^[17]^

### Injury type and location

From the 157 injuries reported, 79% were due to overuse and 21% were traumatic injuries. Arnold and Moody (2018)^[18]^ and Tonoli et al.^[11]^ reported that most of the running-related injuries were muscle, tendon or joint-related injuries. However, the present study reported a higher proportion of muscle and tendon injuries (51%) and a lower proportion of joint-related injuries (28%).

A past research supports the current finding that the knee was the most vulnerable area for injury in long-distance runners.^[18,19]^ Arnold and Moody ^[18]^ reported that knee injuries accounted for 28% of all running-related injuries in runners and that foot/ankle injuries made up the second-largest percentage of injuries (21%–38%). Lopes et al.^[19]^ have also found the most common injury location to be the knee (28%), followed by the foot/ankle (20%). However, they found the spine to be the third most common injury location (13%), whereas this study found it to be uncommon (2%). Logerstedt, Ebert, MacLeod, Heiderscheit, Gabbett and Eckenrode^[20]^ have found that the knee is the most injured joint in sports, due to its position in receiving force from ground-reaction forces and carrying loads from all the body parts above the knee. Over time, the repetitive forces of long-distance running, exacerbated by other factors such as biomechanical deviations and body composition, may damage tissue in and/or around the knee, resulting in pain, reduced performance, and compensation (altered gait). There is large individual variability; however, in the present sample, certain injury risk factors were more prevalent and more significant than others.

### Risk factors for injury

All factors from this study’s questionnaire were analysed for their significance in relation to injury. The significant risk factors identified were a lower BMI (p=0.002), greater height/stature (p=0.05), lack of running experience (p=0.05), not warming up (p<0.001), not cooling down (p=0.01), not stretching before running (p=0.004), not stretching after running (p=0.01), and having suffered a previous injury (p<0.001). Some studies have found higher injury incidence with higher BMI, reasoning that higher BMI places excess load on the musculoskeletal system, resulting in joint-related injuries.^[10]^ However, the present study found contradictory results: lower BMI was associated with a higher risk of injury. A possible reason for this is an inverse concept reported by Tonoli et al.^[11]^ who found that a lower BMI could indicate lower muscle mass, and having less muscle mass may reduce active joint support during running. The contradictory research on BMI suggests no consensus on its effect on injury risk. Although Beard^[10]^ identified greater height as an injury risk factor in runners, it is well-known that previous injury predisposes runner to new or recurrent injury.^[11]^ A previous injury may be associated with altered running biomechanics through compensatory movements. In addition, permanent changes, such as scar tissue formation, may result in decreased tissue elasticity, biomechanical dysfunction, and impaired coordination due to altered neurovascular activity.

The logistic regression model developed from the identified significant injury risk factors (lower BMI, greater height, lack of running experience, not warming up, not cooling down, not stretching before and after running, and previous injury) resulted in an injury prediction model for long-distance runners that was able to correctly predict the incidence of an injury (over a 12-month period) in 77.8% of our sample of runners. This strongly suggests that long-distance runners with significant injury risk factors are 77.8% more likely to sustain an injury. Therefore, this study’s findings may be used in designing injury prevention strategies for South African long-distance runners.

### Limitations and suggestions for future research

This study’s questionnaire was previously used in a Brazilian setting. The questionnaire could have been amended for a South African setting if it were validated by South African researchers. Injury details were self-reported by participants; this could have led to inaccuracies, particularly in the types of injuries sustained. Other self-reported data may also be inaccurate; participants' honesty levels could not be controlled. Although the present study had a sufficient sample size, a larger sample size may have resulted in stronger correlations or additional significant injury risk factors. The types of runners, higher volume versus lower volume runners, that were recruited were not controlled, and this may have resulted in sampling bias. The study’s sample association was also broad in distribution as no affiliation and provincial association characteristics were captured. This study’s sample also consisted of long-distance runners who regularly ran distances between 10 km and 42 km. This wide range of distances could have skewed the statistical variances. Larger sample sizes per running distance category could yield more accurate findings. Finally, the study used a retrospective approach; a prospective methodology may yield different results. Causality was not demonstrated as this study was retrospective and the significant risk factors do not imply cause of injury; a prospective design may yield different outcomes. The retrospective approach may also have introduced recall bias.

## Conclusion

The 12-month injury incidence in runners was high: more than 50% of runners suffered a running-related injury in the previous year; 60% reported new injuries, and 40% reported recurring injuries. There was no significant difference between the male and female runners in terms of injury incidence (p=0.38). The high incidence of injury may be associated, in part, with high training exposure, as the average runner completes more than 40 km per week. Most injuries were overuse injuries (79%), and the three most prevalent injury locations were the knee (26%), the ankle/foot complex (19%), and the lower leg (19%). Using only the significant injury risk factors (lower BMI, greater height, lack of running experience, not warming up, not cooling down, not stretching before and after running, and previous injury), a retrospective linear regression model was developed to explain the probability of being associated to a running-related injury over a 12-month period. The overall prediction accuracy was 77.8%. Therefore, risk factors involving suboptimal training habits, such as warming up and stretching, may contribute to the high injury incidence reported in the study. It is therefore beneficial for runners to take these findings into consideration when developing a hypothesis-generated load management protocol to reduce the risk of injury and re-injury.

## Figures and Tables

**Table 1:** Demographic details for the sample of runners (n=215)

Demographical factors	Sex	Mean ± SD	p-value
Age (years)	Male	44 ± 13	0.92
	Female	44 ± 10	
Weight (kg)	Male	73.8 ± 11.7	<0.001*
	Female	62.9 ± 9.0	
Height (cm)	Male	176.0 ± 7.1	<0.001*
	Female	164.9 ± 8.5	
BMI (kg/m^2^)	Male	23.9 ± 3.7	0.26
	Female	23.3 ± 3.9	

* statistically significant difference (p<0.05)

**Table 2:** Participants’ running experience (n=215)

Running experience	Male runners (%)	Female runners (%)	Total % sample
Novice (*running for 6 months to 2 years*)	30	25	28
Experienced (*running for longer than 2 years*)	70	75	72

**Table 3:** Training frequency, distance, speed and duration (n=215)

Running characteristics	Males	Females	Total group	p-value
Average training frequency per week	4.2	4.3	4.3	0.17
Average training distance (km/week)	42	39	40	0.19
Average running speed (min/km)	5.58	6.14	5.86	0.04*
Average training duration (min)	68	68	68	0.85

* statistically significant difference (p<0.05)

**Table 4:** Injury details of South African long-distance runners (n=215)

Injury factor	Males (% total group)	Females (% total group)	Total group (%)	p-value
Overall incidence *	54	48	51	0.38
New injury	29	31	60	
Recurring injury	21	19	40	
**Type of injury**				
Overuse	43	36	79	0.58
Traumatic	10	11	21	
**Injury severity**				
Minor	15	14	29	0.13
Moderate	34	24	58	
Severe	4	8	12	
Critical (hospitalised)	1	0	1	
**Injury locations**				
Knee	14	12	26	0.84
Ankle/foot complex	10	9	19	
Lower leg	10	9	19	
Hip/pelvis	7	7	14	
Thigh	8	4	12	
Calf	4	3	7	
Spine	1	1	2	
Upper limbs	1	0	1	

Data presented as percentages.

* Overall incidence compares to the total sample while new and recurring injuries compares to the injured group.

**Table 5:** Injury risk factors for long-distance runners (n=215)

Characteristics	Association (Pearson Chi Square)	p-value	Cramer’s V (effect size)
Type of work	1.623	0.2	0.087
Alcohol use	4.419	0.22	0.143
Tobacco use	0.889	0.35	0.064
Suffering from a chronic condition	0.092	0.76	0.021
Use of medication	0.055	0.82	0.033
**Training characteristics**			
Training frequency	1.514	0.68	0.085
Types of races completed	7.152	0.31	0.182
Not using a running coach	1.094	0.3	0.071
Participation in other sports	1.564	0.21	0.085
Running motivation	4.462	0.11	0.144
Lack of running experience (novice runner)	4.019	0.05*	0.137
Not stretching before running	11.235	0.004*	0.229
Not stretching after running	9.181	0.01*	0.207
Not warming up	18.274	<0.001*	0.293
Not cooling down	9.310	0.01*	0.208
Time period for shoe replacement	4.144	0.25	0.139
Reason for shoe replacement	4.863	0.09	0.151
Shoes with special features	3.092	0.21	0.120
Shoes with additional cushioning	0.681	0.41	0.056
**History of previous injury**			
Suffered a previous injury (prior to 12 months ago)	23.280	<0.001*	0.347

* Statistically significant injury risk factor.

Degrees of freedom (df) = 213.
